# Hydrosalpinx perturbs the tubal and endometrial immune environment

**DOI:** 10.1530/RAF-25-0204

**Published:** 2026-06-26

**Authors:** Fatima Aljassim, Charlotte H Rigby, Josephine A Drury, Rafah Alnafakh, Corie Rushworth, Alison Maclean, Dharani K Hapangama, Christopher J Hill

**Affiliations:** ^1^ Centre for Women’s Health Research, Department of Women’s and Children’s Health, Institute of Life Course and Medical Sciences, University of Liverpool, Member of Liverpool Health Partners, Liverpool, UK; ^2^ Imam Abdulrahman Bin Faisal University, Dammam, Saudi Arabia; ^3^ Liverpool Women’s Hospital NHS Foundation Trust, Member of Liverpool Health Partners, Liverpool, UK

**Keywords:** hydrosalpinx, fallopian tube, immune cells, endometrium, infertility

## Abstract

**Abstract:**

Hydrosalpinx is a chronic pathological manifestation seen in approximately 30% of tubal disease cases. In addition to contributing to infertility and pelvic pain, hydrosalpinx has been associated with early pregnancy loss and reduced implantation in assisted reproductive technology. Though not fully understood, previous studies have demonstrated altered immunological activity in those with hydrosalpinx, which has been suggested to impact endometrial receptivity. To validate these findings and to explore the pathogenesis of tubal disease, the aim of this study was to identify and quantify the immune cell population in tubes with hydrosalpinx and in the endometrium from the same patients. This observational study included patients in the secretory phase of the menstrual cycle undergoing salpingectomy/salpingo-oophorectomy with/without hysterectomy for benign gynaecological conditions. The number and type of immune cells present in endometrial and fallopian tube tissue sections were investigated using immunostaining for CD45, CD3, CD4, CD8, CD56 and CD68. We show that T cells are more abundant in the endometrium of women with hydrosalpinx and that cytotoxic T cell numbers are lower in diseased tubes. Uterine natural killer cells and macrophages are significantly elevated in both tubal and endometrial tissues in hydrosalpinx, demonstrating a disruption in the normal uterine immune cell microenvironment that may affect endometrial receptivity, with implications for tubal disease-associated subfertility.

**Lay summary:**

Hydrosalpinx is a common condition characterised by inflammation, distortion and fluid accumulation in the fallopian tubes. Hydrosalpinx is a leading cause of subfertility; however, the mechanisms that cause this problem are not fully understood. We studied the immune cells present in both the fallopian tubes and uterine lining (endometrium) of women with hydrosalpinx and compared them to samples from women without tubal disease. The samples were collected in the second half of the menstrual cycle, when the uterine lining is prepared for implantation of a fertilised embryo. We quantified the total immune cell numbers and specific immune cell subtypes (these are immune cells with specific roles in the body, such as destroying harmful bacteria or regulating the immune system) in the different tissues and compared them between the hydrosalpinx and control groups. We found that hydrosalpinx is associated with both tubal and endometrial immune cell changes, which may contribute to infertility by affecting the normal physiological processes that allow the endometrium to accept an embryo and establish a pregnancy.

## Introduction

The fallopian tubes (FTs) are a pair of narrow, muscular conduits that provide a critical connection between the ovaries and uterus in the female reproductive system (Han & Sadiq 2025). Through their involvement in many physiological processes, such as gamete transportation, zygote passage and pre-implantation embryonic development, the FTs play a fundamental role in fertility (Croxatto 2002, Maclean *et al.* 2020, Han & Sadiq 2025). Consequently, tubal pathologies have a detrimental effect on reproductive function, with tubal disease thought to account for up to 25% of all cases of infertility (Ng & Cheong 2019, Dutton *et al.* 2025).

Hydrosalpinx, defined as tubal distension accompanied with fluid accumulation, is a chronic pathological manifestation seen in approximately 30% of tubal disease cases (Blazar *et al.* 1997, Aboulghar *et al.* 1998, Ng & Cheong 2019). Hydrosalpinx initiation stems from tubal occlusion, predominantly in the distal fimbriated region, which is mainly caused by pelvic inflammatory disease (PID), although endometriosis, pelvic adhesions and previous surgery are known to promote disease development (Puttemans & Brosens 1996, Ng & Cheong 2019). In addition to contributing to infertility, hydrosalpinx is associated with early pregnancy loss and reduced implantation success in assisted reproductive technology, such as *in vitro* fertilisation (IVF), with success rates reportedly reduced by up to 50% (Vandromme *et al.* 1995, Zeyneloglu *et al.* 1998). Though not fully understood, several studies have proposed that an unfavourable intrauterine environment is created by hydrosalpinx fluid transfer, which disrupts endometrial receptivity and restricts pregnancy establishment (Strandell *et al.* 1994, Vandromme *et al.* 1995). As a result, following diagnosis via a hysterosalpingogram, current NICE guidance recommends that all women with hydrosalpinges be offered a laparoscopic salpingectomy prior to IVF treatment to improve the likelihood of a live birth (NICE guideline 2026).

The inflammatory response associated with hydrosalpinx has been investigated by limited studies; increased numbers of T lymphocytes, in particular CD4^+^ helper T cells, and macrophages have been observed in hydrosalpinx relative to healthy tubes (Haney *et al.* 1983, Edelstam & Karlsson-Parra 1996, Kinnunen *et al.* 2000, Rigby *et al.* 2022). A study by Copperman *et al.* demonstrated that the endometrium exhibits a localised inflammatory reaction in response to hydrosalpingeal fluid, with significant leucocyte recruitment and interleukin-2 elevation compared with those with healthy FTs. Increased immunological activity in hydrosalpinx has been suggested to contribute to decreased reproductive functions (Copperman *et al.* 2006). However, more granular details of hydrosalpinx-induced immunological changes in both tubal and endometrial environments are not available but are required to elucidate disease aetiology and pathophysiology. These findings will facilitate the development of novel fertility treatments that avoid surgical intervention. Therefore, the objective of this study was to comprehensively phenotype the immune cell population in hydrosalpinx with matched endometrium from the same patient.

## Materials and methods

### Ethics for the collection and use of human tissue

The use of human tissue was approved by the Liverpool Adult Research Ethics Committee (24/WA/0296 and 19/SC/0449).

### Study population

A total of 18 participants were included in this study. FT (distal portion) and endometrial biopsies were collected from women undergoing salpingectomy/salpingo-oophorectomy with/without hysterectomy for benign gynaecological conditions at Liverpool Women’s Hospital NHS Foundation Trust between 2010 and 2023. All participants were in the secretory phase of the menstrual cycle and were not on hormonal treatments for at least three months preceding sample collection. The control group (*n* = 7) were patients with no tubal abnormalities. The presence of hydrosalpinx (*n* = 11) was identified by pre-operative imaging, verified intraoperatively by an experienced gynaecologists and subsequently confirmed by an experienced histopathologist. Matched tubal and endometrial tissue was available for all control and seven hydrosalpinx participants; the remaining four participants in the disease group provided tubal tissue only. The endometrial cycle phase was assigned with the patients’ last menstrual period and confirmed by an experienced histopathologist. Demographic information of the study population is presented in Table 1.

**Table 1:** Participant demographics. BMI, body mass index; IQR, interquartile range.

	Control	Hydrosalpinx	*P*-value
Number	7	11	
Age in years, median (IQR)	39 (11)	38 (17)	0.68*
BMI (kg/m^2^), median (IQR)	29.6 (8.2)	29.1 (7.5)	0.75*
Smoker, *n* (%)	1 (14)	1 (9)	1.00^†^
Nullipara, *n* (%)	1 (14)	6 (55)	0.15^†^
Infertile, *n* (%)	1 (14)	7 (64)	0.07^†^
Endometriosis, *n* (%)	2 (29)	2 (27)	1.00^†^
Adenomyosis, *n* (%)	2 (29)	0 (0)	0.14^†^
Leiomyoma, *n* (%)	2 (29)	1 (9)	0.53^†^

* Mann–Whitney U test.

^†^ Fisher’s exact test.

### Immunohistochemistry

Immunohistochemical procedures were performed on 3 μm sections of formalin-fixed paraffin-embedded (FFPE) tissue as described previously (Al-Lamee *et al.* 2022). In brief, tissue sections were deparaffinised in xylene and rehydrated through a gradient of ethanol. For restoration of protein epitopes, heat-induced antigen retrieval was performed by pressure cooking sections in 10 mM citrate buffer pH 6.0. Tissue was blocked with 2.5% [v/v] normal horse serum followed by primary antibody incubation (Supplementary Table 1 (see section on Supplementary materials given at the end of the article)). For chromogenic immunostaining, sections were incubated with the appropriate ImmPRESS® polymer (Supplementary Table 1) for 30 min followed by ImmPACT® DAB chromogen or ImmPACT Vector Red Substrate (Vector Laboratories, UK) for 10 min or 30 min, respectively. The brown/red positive staining was counterstained with Gill II haematoxylin (Thermo Fisher Scientific, UK), following which sections were dehydrated, cleared with xylene and mounted with Consul-Mount (Thermo Fisher Scientific). For immunofluorescence staining, secondary antibodies AlexaFluor 488 anti-rabbit and AlexaFluor 594 anti-mouse (Cell Signaling Technology, USA) were applied at 1:000 dilution and incubated for 2 h. To reduce autofluorescence, the TrueVIEW® Autofluorescence Quenching Kit (Vector Laboratories) was applied as per the manufacturer’s instructions. Sections were mounted with VECTASHIELD® mounting medium with DAPI (Vector Laboratories). Images were captured using a Zeiss LSM 800 confocal laser scanning microscope equipped with an Axiocam 506 monochrome camera (Zeiss, Germany).

### Staining quantification

Sections stained with DAB chromogen were digitalised using an Aperio CS2 Slide Scanner (Leica Biosystems, Germany) and visualised with Aperio ImageScope software (version 12.4.3). Four random regions of interest (ROIs) at maximum magnification (40×) were examined for each cellular compartment of FT (epithelium and stroma) and endometrium (luminal epithelium, functionalis glands and functionalis stroma) tissue sections. For each ROI, background was removed and the total number of cells was calculated using the StarDist plugin for FIJI/ImageJ (Schindelin *et al.* 2012, Schmidt *et al.* 2018). Positive cells were counted manually for each marker, and the average percentage of positive cells was calculated across the four ROIs for each cellular compartment. For some samples, specific tissue regions (e.g. luminal epithelium) were missing or insufficient for quantification. Data for all samples and markers are given in Supplementary Table 2.

### Statistical analysis

Non-parametric statistical tests were performed using GraphPad Prism, version 5.0 (Mann–Whitney U test and Fisher’s exact test). Results were considered statistically significant when *P* < 0.05 (*), *P* < 0.01 (**) and *P* < 0.001 (***). Box and whisker plots show the median value (horizontal line), 25th and 75th percentiles (box) and data range (whiskers). Data points represent the average positive cell count across the four ROIs for each cellular compartment.

## Results

### Participant demographics

Eighteen participants aged 24–49 years were recruited to this study and divided into two groups: the study group (*n* = 11) diagnosed with hydrosalpinx and the control group (*n* = 7) with no tubal pathology. The population was predominantly non-smokers, except for one individual in each group. Participants had a parity ranging from 0 to 5 and body mass index (BMI) values between 16.8 and 37.6. Infertility was common in the hydrosalpinx group, affecting 64% of women, compared with 14% in the control group.

Chronic gynaecological conditions, including endometriosis, adenomyosis and uterine fibroids, were present across both groups, although adenomyosis was not reported in the hydrosalpinx group. No statistically significant differences in demographic characteristics were observed between the two groups (Table 1).

### Immune cell populations in normal fallopian tubes and matched endometrium compared to hydrosalpinx in the secretory phase

**Figure 1:**
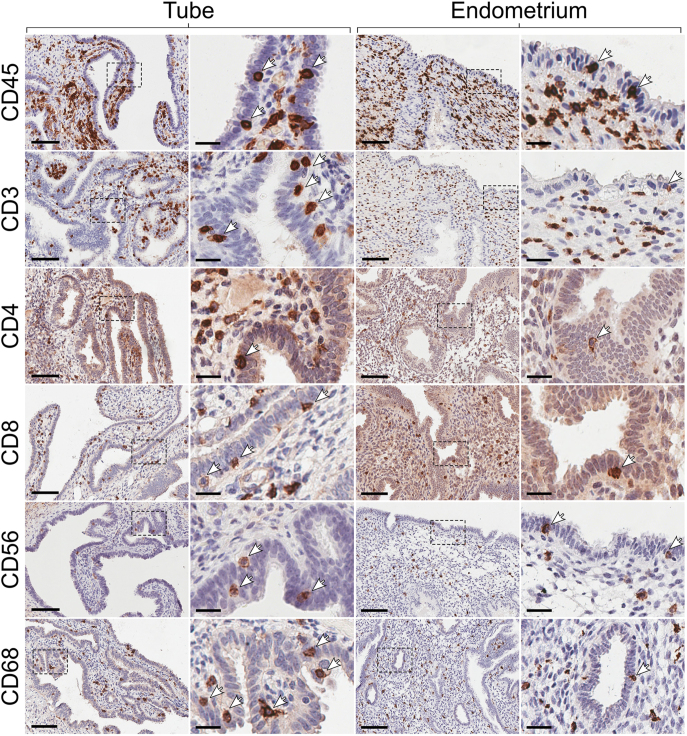
Immunohistochemical staining of immune cell populations in the FTs and endometrial functionalis. Micrographs show representative immunostaining for CD45, CD3, CD4, CD8, CD56 and CD68. Zoomed views of boxed areas are shown to the right of each image. Intraepithelial leucocytes are indicated by white arrows. The scale bars are 90 and 20 μm for low- and high-power images, respectively.

**Figure 2:**
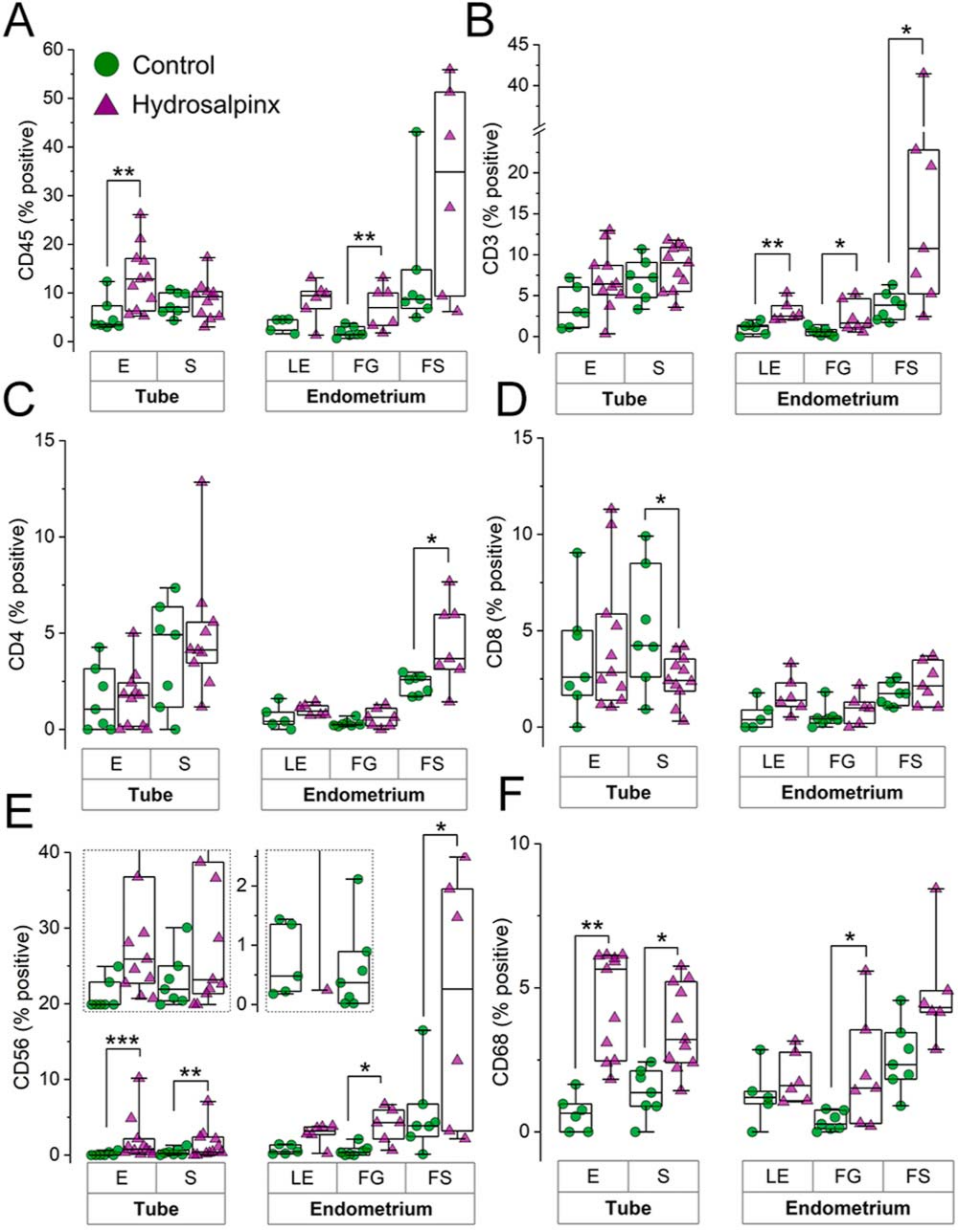
Immune cell marker expression in healthy and damaged FTs with matched endometrium. The box plots represent the average percentage of cells expressing CD45 (A), CD3 (B), CD4 (C), CD8 (D), CD56 (E) and CD68 (F) in the FT and endometrium tissue sections. The boxed regions in panel E show low-value data points on a magnified scale. Positive cells were quantified in tubal epithelium (E) and stroma (S), and endometrial luminal epithelium (LE), functionalis glands (FG) and functionalis stroma (FS).

Leucocyte populations were assessed in tissue sections of the FTs and matched endometrium in the control and hydrosalpinx groups (Fig. 1). All immune cell markers investigated (CD45 – pan-leucocyte marker; CD3 – pan-T cell marker; CD4 – helper T cell marker; CD8 – cytotoxic T cell marker; CD56 – uNK cell marker; and CD68 – pan-macrophage marker) were present in the stromal and epithelial (intraepithelial leucocytes) compartments of the FTs and matched endometrium. One of the most striking features was the markedly elevated intraepithelial CD45^+^ leucocytes observed in both the tubal and endometrial epithelium of participants diagnosed with hydrosalpinx compared with the control group (Fig. 2A).

### Adaptive immunity

CD3^+^ T cell abundance was comparable in the healthy and damaged FTs but was significantly increased in the matched endometrial luminal epithelium, glands and stroma in the hydrosalpinx group (Fig. 2B). CD4^+^ helper T cell numbers were comparable in both healthy and pathological FTs but elevated in the endometrial stromal compartment of the hydrosalpinx group (Fig. 2C). CD8^+^ cytotoxic T cell abundance was significantly decreased in the FT stroma of the hydrosalpinx group relative to controls but was comparable across all endometrial compartments (Fig. 2D).

### Innate immunity

Uterine natural killer (uNK) cells expressing CD56 were significantly more abundant in the FT epithelium and stroma in the hydrosalpinx group, whilst also increasing in the patient-matched endometrial glands and stroma relative to controls (Fig. 2E). CD68^+^ macrophages were significantly more numerous in the FT epithelium and stroma of hydrosalpinx and in the functionalis glands of patient-matched endometrium (Fig. 2F).

### Increased abundance of proliferative leucocytes in hydrosalpinx tubes

**Figure 3:**
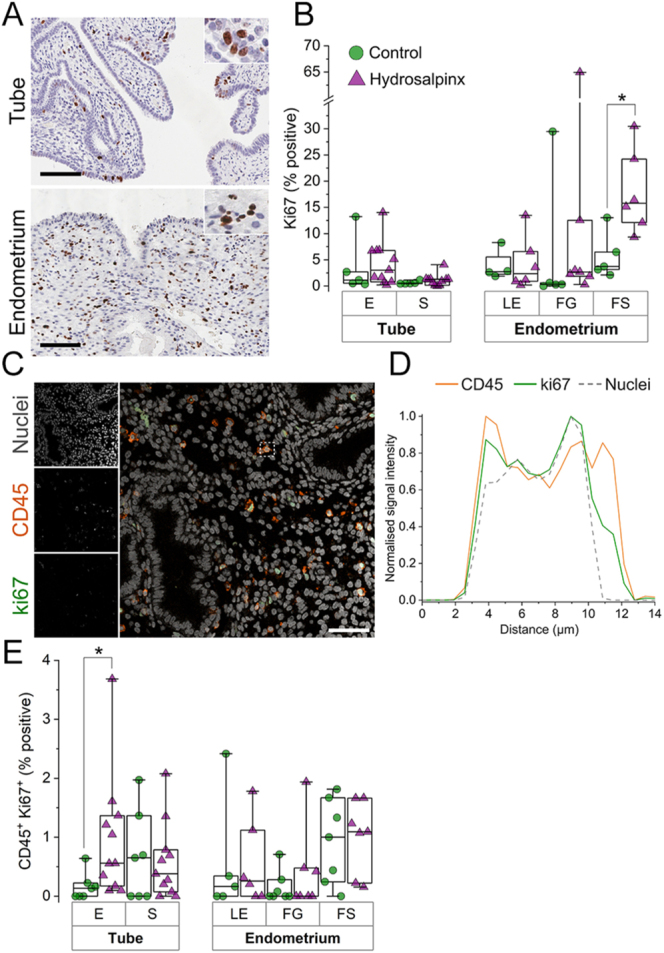
Cellular proliferation in healthy and damaged tubes with matched endometrium. (A) Representative micrographs show immunostaining for Ki67. Zoomed regions are highlighted in each panel. Scale bar = 90 μm. (B) Average number of Ki67^+^ cells in tubal epithelium (E) and stroma (S), and endometrial luminal epithelium (LE), functionalis glands (FG) and functionalis stroma (FS). (C) Representative micrograph of CD45 (orange) and Ki67 (green) in the endometrial functionalis. Scale bar = 50 μm. (D) Fluorescence intensity line scan profile generated from the boxed cell in the merged image. (E) Average number of dual CD45^+^ Ki67^+^ cells across tissue regions.

Actively proliferating cells were quantified in FT and endometrial tissue sections (Fig. 3A). The number of Ki67^+^ cells in the FTs was comparable between the control and hydrosalpinx cohorts. In the matched endometrium, proliferative cell numbers in the stromal compartment, but not epithelial compartments, were significantly higher in the hydrosalpinx cohort (Fig. 3B). Co-expression of CD45 and Ki67 revealed a population of proliferative leucocytes in both tubal and endometrial tissues (Fig. 3C, D, Supplementary Fig. 1). The number of CD45^+^ Ki67^+^ leucocytes in endometrial tissue was comparable between study groups (Fig. 3E). However, CD45^+^ Ki67^+^ leucocytes were significantly more abundant in the tubal epithelium of diseased versus healthy FTs (Fig. 3E).

## Discussion

This study aimed to provide a broad characterisation of immune cell populations in both the FTs and corresponding endometrial luminal epithelium and functionalis of women with hydrosalpinx, compared with a healthy control population. Through immunohistochemical analysis, we identified a distinct range of immune cells from both the innate and adaptive immune systems. To the best of our knowledge, this is the first study to immunohistochemically characterise the immunological differences between the FTs and matched endometrium in normal and hydrosalpinx conditions. Elevated immunological activity was observed in both tubal and endometrial tissues in participants with hydrosalpinx, with significant increases in the expression of CD3, CD4, CD45, CD56 and CD68. Notably, CD8 expression was significantly lower in the tubal stroma of diseased versus healthy FTs, suggesting differential immune responses between the two conditions.

In the healthy FTs, T lymphocytes represent the dominant immune cell population, accounting for approximately 40–60% of all leucocytes (Ardighieri *et al.* 2014, Rigby *et al.* 2022, Visnyaiová *et al.* 2024). Among T lymphocytes, CD8^+^ cytotoxic T cells were the most abundant subset. CD4^+^ helper T cells were more scarcely distributed and sometimes absent, though when present, most often localised within the lamina propria (Boehme & Donat 1992, Rigby *et al.* 2022).

Although the inflammatory response associated with hydrosalpinx has previously been reported, only limited exploration into the types and distribution of immune cells has been published. Here, we elucidate the immunophenotype of tubal tissue affected by hydrosalpinx, which exhibits an elevated leucocyte population predominantly localised to the tubal epithelium and enhanced leucocyte proliferation. Hydrosalpinx tubes are associated with significant immune cell infiltration compared with healthy counterparts (Rigby *et al.* 2022). This condition particularly involves disruptions in the complement system and phagocytosis pathways (Yohannes *et al.* 2019). Analyses of tubal specimens have demonstrated a marked increase in inflammatory cells, including macrophages, lymphocytes, plasma cells and neutrophils, in hydrosalpinx-affected tubes (Copperman *et al.* 2006). Furthermore, single-cell RNA sequencing has revealed alterations in immune cell populations, suggesting a heightened inflammatory environment in hydrosalpinx (Ulrich *et al.* 2022). In this study, no significant difference in the abundance of CD3^+^ or CD4^+^ T cells in hydrosalpinx compared with healthy FTs was observed. In contrast, CD8^+^ T cells were significantly less abundant in hydrosalpinx tubal stroma compared with healthy tubes. T cells, particularly CD8^+^ cytotoxic T cells, are the most abundant immune cells in healthy FTs (Ardighieri *et al.* 2014, Rigby *et al.* 2022). CD8^+^ lymphocytes play a significant role in the human FTs, being involved in local immunity and potentially contributing to immune tolerance for normal reproductive processes (Boehme & Donat 1992). CD8^+^ T cells may facilitate immune tolerance, allowing for the transport of sperm and embryos without triggering local immune responses (Kutteh *et al.* 1990, Boehme & Donat 1992). Edelstam & Karlsson-Parra reported a higher number of CD4^+^ T cells in sactosalpinx and pelvic adhesion FT biopsies compared with CD8^+^ T cells in normal FT biopsies. This shift may result from local inflammatory factors within the tissue and could partly explain the reduced fertility observed in these patients, even when the FTs are patent (Edelstam & Karlsson-Parra 1996).

Populations of CD68^+^ macrophages and CD56^+^ uNK cells were observed in hydrosalpinx tissue, with both exhibiting highly significant increases in the tubal epithelium and stroma when compared with healthy tubes. These increases suggest an upregulation in the activation of the innate immune system, potentially stimulated by the accumulation of hydrosalpingeal fluid in the tubal lumen, which has previously been shown to cause direct tissue toxicity (Bedaiwy *et al.* 2005). Tubal macrophage numbers vary throughout the menstrual cycle and increase during the progesterone-dominant secretory phase (Rigby *et al.* 2022). This fluctuation highlights their role in preparing the FT environment for oocyte transport and fertilisation. Indeed, macrophages produce prostaglandins that can influence tubal motility and facilitate gamete transport (Safwat *et al.* 2008, Rigby *et al.* 2022). However, prostaglandin levels in healthy FTs relative to hydrosalpinx have not been investigated. Increased macrophage activity is linked to hydrosalpinx, potentially contributing to tissue destruction and playing a role in tubal pathologies that can lead to infertility (Gaytán *et al.* 2007, Rigby *et al.* 2022, Ulrich *et al.* 2022).

As both the FTs and uterus share embryological derivation from the Müllerian duct, the tubal lumen exists as a continuation of the uterine cavity (Hill *et al.* 2020). Consequently, some gynaecological pathologies have been identified to spread between both tissues; up to 30% of patients with endometriosis are reported to have tubal involvement (Rezvani & Shaaban 2011, Foti *et al.* 2016). Endometrial exposure to hydrosalpingeal secretions is known to induce inflammatory pathways and perturb endometrial receptivity. Copperman *et al.* observed a localised endometrial inflammatory reaction mediated via interleukin-2 that correlated with the presence of hydrosalpingeal fluid in the uterine cavity (Copperman *et al.* 2006). This inflammatory reaction has been thought to contribute to altered endometrial receptivity; Daftary & Taylor identified a decrease in endometrial *HOXA10* mRNA expression, a transcription factor found to have an essential role in implantation in the murine model, under increasing concentrations of hydrosalpingeal fluid, suggesting an underlying mechanism for diminished implantation (Daftary & Taylor 2002). We observed significant elevations in the endometrial expression of CD3-, CD4-, CD45-, CD56- and CD68-positive cells of those with hydrosalpinx. T cells constitute 5–20% of decidual lymphocytes, whilst CD4^+^ helper T cells and regulatory T cells are crucial for the establishment of immune tolerance during the window of implantation (Lee *et al.* 2011, Ma *et al.* 2022). Indeed, T cell imbalance and functional insufficiency have been linked to infertility, miscarriage and pre-eclampsia (Guerin *et al.* 2009). Endometrial macrophage and uNK cell numbers increase during the secretory phase, where they play many important roles to facilitate endometrial receptivity and pregnancy establishment, including tissue remodelling, angiogenesis, stromal decidualisation and a controlled inflammatory response. Imbalances in endometrial macrophage polarisation and retention have been linked to recurrent implantation failure (RIF) and recurrent pregnancy loss (Ma *et al.* 2025, Park *et al.* 2025). Similarly, changes in endometrial uNK cell abundance have been linked to infertility, with some studies demonstrating an increase in women with RIF (Marron *et al.* 2019, Marron & Harrity 2019).

Taken together, the observed increase in endometrial leucocyte abundance in women with hydrosalpinx suggests a direct impact of tubal disease on the uterine immune environment, which may affect fertility. Future research should explore the relationship between endometrial immune cell perturbations and known hydrosalpinx-induced changes in receptivity markers (Savaris & Giudice 2007) to further dissect the pathological mechanisms of tubal disease-associated subfertility. Furthermore, it will be important to characterise the underlying cause of hydrosalpinx in future cohorts, as different bacterial pathogens may elicit unique changes to the tubal and endometrial immune cell profiles.

## Supplementary materials





## Declaration of interest

The authors declare that there is no conflict of interest that could be perceived as prejudicing the impartiality of the work reported.

## Funding

This work was supported by Imam Abdulrahman Bin Faisal University, Saudi Arabia 30199 (FA), the University of Liverpool (CHR), Wellbeing of Women RG2137 (CJH and DKH), the Vinehill Trust (CJH) and a Medical Research Council Grant MR/V007238/1 (AM and DKH).

## Author contribution statement

DKH and CJH conceived the study. FA, CHR, RA, CR and AM performed experiments. FA, CR and CJH analysed data and prepared figures. FA, CJH and DKH wrote the first draft of the manuscript. All authors finalised, critically appraised and approved the final version.
