# Supplementary material for: Hydrosalpinx perturbs the tubal and endometrial immune environment
Source: Reprod Fertil. 2026 Jun 26;7(2):RAF250204. doi: 10.1530/RAF-25-0204 (PMC13317832; doi:10.1530/RAF-25-0204)
Supplement: Supplementary file 1 [file supplementary_materials.pdf]

Supplementary information

Supplementary Table 1. Primary antibodies used for immunohistochemistry. Abbreviations: alkaline phosphatase (AP), cluster of differentiation (CD), heat-induced antigen retrieval (HIAR), horse radish peroxidase (HRP).

Target	Host	Clone	HIAR	Dilution	Incubation	Secondary	Supplier
CD3	Rabbit	301	3 min, citrate pH 6.0	1:500	16 h 4 °C	Horse anti-rabbit HRP	Sino Biological
CD4	Mouse	23	3 min, citrate pH 6.0	1:500	16 h 4 °C	Horse anti-mouse HRP	Sino Biological
CD8	Mouse	38	3 min, citrate pH 6.0	1:500	16 h 4 °C	Horse anti-mouse HRP	Sino Biological
CD45	Mouse	2B11, PD7/26	3 min, citrate pH 6.0	1:100	16 h 4 °C	Horse anti-mouse HRP<br>Horse anti-mouse AP<br>Goat anti-rabbit AlexaFluor 594	Dako

CD56	Mouse	1B6	3 min, citrate pH 6.0	1:50	16 h 4 °C	Horse anti-mouse HRP	Novocastra
CD68	Mouse	PG-M1	3 min, citrate pH 6.0	1:1000	16 h 4 °C	Horse anti-mouse HRP	Dako
Ki67	Mouse	MM1	4 min, citrate pH 6.0	1:200	16 h 4 °C	Horse anti-mouse HRP	Novocastra
Ki67	Rabbit	SP6	4 min, citrate pH 6.0	1:100	16 h 4 °C	Horse anti-rabbit HRP Goat anti-rabbit AlexaFluor 488	Abcam

Supplementary Table 2. Individual datapoints for all participants and markers quantified across tubal and endometrial tissue.

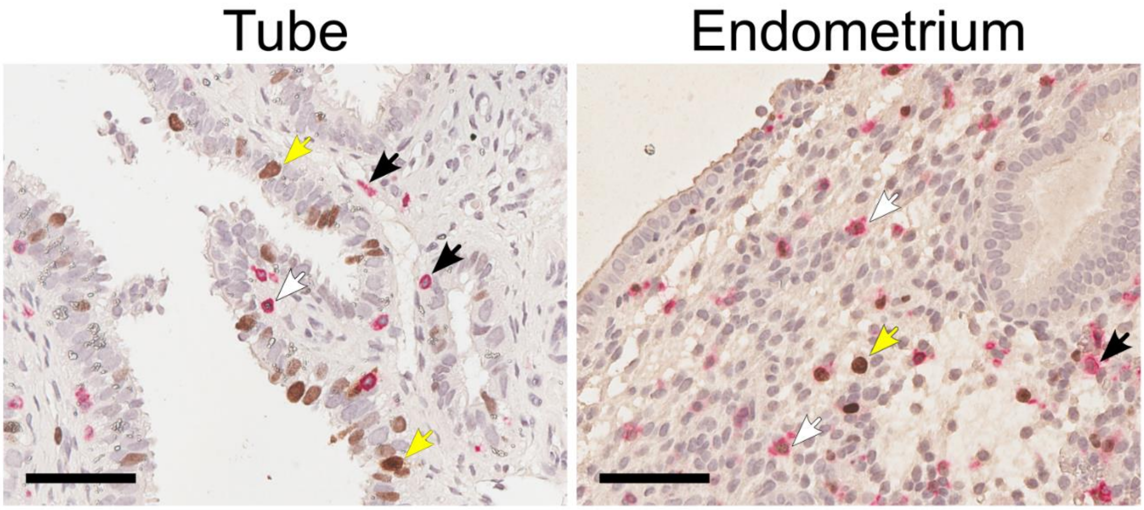

Supplementary Figure 1. Immunohistochemical staining of leucocytes and proliferative cells in the fallopian tubes and endometrial functionalis. Micrographs show representative dual chromogenic staining for CD45 (pink) and Ki67 (brown). Dual stained cells (white arrows), $CD45^{+}$ $Ki67^{-}$ (black arrows) and $CD45^{-}$ $Ki67^{+}$ (yellow arrows) are highlighted. Scale bars = 60 μm.
